# Highly Conformal Craniospinal Radiotherapy Techniques Can Underdose the Cranial Clinical Target Volume if Leptomeningeal Extension through Skull Base Exit Foramina is not Contoured

**DOI:** 10.1016/j.clon.2017.02.013

**Published:** 2017-07

**Authors:** D.J. Noble, T. Ajithkumar, J. Lambert, I. Gleeson, M.V. Williams, S.J. Jefferies

**Affiliations:** ∗ Cancer Research UK VoxTox Research Group, Department of Oncology, University of Cambridge, Cambridge Biomedical Campus, Addenbrooke's Hospital, Cambridge, UK; † Department of Oncology, Cambridge University Hospital's NHS Foundation Trust, Cambridge, UK; ‡ West German Proton Therapy Centre Essen, Essen, Germany; § Medical Physics Department, Cambridge University Hospital's NHS Foundation Trust, Cambridge, UK

**Keywords:** Craniospinal radiotherapy, intensity-modulated radiotherapy, medulloblastoma, proton beam therapy, radiotherapy planning

## Abstract

**Aims:**

Craniospinal irradiation (CSI) remains a crucial treatment for patients with medulloblastoma. There is uncertainty about how to manage meningeal surfaces and cerebrospinal fluid (CSF) that follows cranial nerves exiting skull base foramina. The purpose of this study was to assess plan quality and dose coverage of posterior cranial fossa foramina with both photon and proton therapy.

**Materials and methods:**

We analysed the radiotherapy plans of seven patients treated with CSI for medulloblastoma and primitive neuro-ectodermal tumours and three with ependymoma (total *n* = 10). Four had been treated with a field-based technique and six with TomoTherapy™. The internal acoustic meatus (IAM), jugular foramen (JF) and hypoglossal canal (HC) were contoured and added to the original treatment clinical target volume (Plan_CTV) to create a Test_CTV. This was grown to a test planning target volume (Test_PTV) for comparison with a Plan_PTV. Using Plan_CTV and Plan_PTV, proton plans were generated for all 10 cases. The following dosimetry data were recorded: conformity (dice similarity coefficient) and homogeneity index (D_2_ − D_98_/D_50_) as well as median and maximum dose (D_2%_) to Plan_PTV, V_95%_ and minimum dose (D_99.9%_) to Plan_CTV and Test_CTV and Plan_PTV and Test_PTV, V_95%_ and minimum dose (D_98%_) to foramina PTVs.

**Results:**

Proton and TomoTherapy™ plans were more conformal (0.87, 0.86) and homogeneous (0.07, 0.04) than field-photon plans (0.79, 0.17). However, field-photon plans covered the IAM, JF and HC PTVs better than proton plans (*P* = 0.002, 0.004, 0.003, respectively). TomoTherapy™ plans covered the IAM and JF better than proton plans (*P* = 0.000, 0.002, respectively) but the result for the HC was not significant. Adding foramen CTVs/PTVs made no difference for field plans. The mean D_min_ dropped 3.4% from Plan_PTV to Test_PTV for TomoTherapy™ (not significant) and 14.8% for protons (*P* = 0.001).

**Conclusions:**

Highly conformal CSI techniques may underdose meninges and CSF in the dural reflections of posterior fossa cranial nerves unless these structures are specifically included in the CTV.

## Highlights

• Highly conformal techniques can deliver craniospinal radiotherapy for medulloblastoma.
• Meninges and cerebrospinal fluid extend through the skull base with cranial nerves.
• These structures receive inadequate dose if they are not specifically contoured.
• Posterior fossa foramina should be included in the craniospinal clinical target volume.

## Introduction

High-quality radiotherapy remains an important treatment for patients with medulloblastoma [1]. The literature reports a relationship between inadequate technique and patterns of relapse [2], [3], [4]. Although most of these tumours arise in the posterior cranial fossa, they are known to spread to leptomeningeal surfaces throughout the craniospinal axis (CSA) and the evolution of radiotherapy techniques used for medulloblastoma reflects this biology. Current standard practice includes a moderate dose of craniospinal irradiation (CSI), followed by a boost to the tumour bed in the posterior fossa, where historical data suggest that the risk of relapse is higher [5], [6].

There has been rapid development in both technology and treatment technique for medulloblastoma. The classical ‘field-based’ approach, whereby a parallel pair of opposed lateral photon fields are matched to the divergence of a posterior field is still used by many centres. Others have adopted three-dimensional conformal and intensity-modulated radiotherapy (IMRT) approaches [7]. Helical arc IMRT delivery offers an elegant solution to many of the technical challenges posed by CSI [8], [9]. The potential of proton therapy for medulloblastoma has been considered for two decades [10], [11]. It has attractive advantages, especially in children, with the possibility of reduced dose to critical organs at risk (OARs) and a lower whole body integral dose [12], [13].

Studies report a high risk of medulloblastoma recurrence in the cribriform plate and inferior frontal and temporal lobes when they were not included in the target volume or when they were missed due to shielding of the eyes [4], [14], [15], [16]. These structures are therefore now included routinely in the clinical target volume (CTV).

With the evolution of volume-based radiotherapy techniques, an unresolved issue is whether meningeal reflections of cranial nerves as they exit their respective skull base foramina should be included in the CTV. Field-based CSI protocols recommend a margin of 5 mm below the cribriform plate and at least 10 mm below the skull base elsewhere in order to cover cranial nerve meningeal reflections. Recently published work has shown that cerebrospinal fluid (CSF) and thus surrounding meningeal surfaces may be found over a centimetre beyond the internal table of the skull in the internal acoustic meatus (IAM), jugular foramen (JF) and hypoglossal canal (HC) [17]. This finding may be of interest for clinicians planning radiotherapy for patients with medulloblastoma.

The purpose of this study was to test the hypothesis that modern, volume-based conformal radiotherapy techniques underdose meningeal surfaces in the posterior fossa unless these structures are specifically included in the CTV.

## Materials and Methods

This project was registered and approved as a service evaluation (Proposal No. 193) with the local oncology directorate and audit department.

### Patient Datasets

Patients treated with CSI for primitive neuro-ectodermal tumours at our institution between 2005 and 2014 were identified (*n* = 7). Another who received CSI after locoregional recurrence of ependymoma was identified, and to further increase the sample size, two patients who underwent focal, posterior fossa radiotherapy for primary ependymoma were also included. In both cases, the CTVs were large, covering much of the posterior fossa, and were constrained by the internal table of the skull. Importantly, the CTV margins used included the skull base foramina and extended beyond this constraint, posing the same dosimetric question as seen with the eight CSI cases. There were, therefore, 10 cases in total. All patients had been immobilised with a thermoplastic shell and undergone computed tomography simulation (3 mm slices). Details of patient characteristics, disease, treatment technique, dose and platform are all given in Table 1.

**Table 1:** Case details

Case no.	Age	Year treated	Diagnosis	Clinical target volume assessed∗	Dose/regimen	Technique and platform
1	5	2005	Medulloblastoma	Phase I whole CSA + phase II post-fossa boost	62 Gy in 40 fractions (bd fractions)	Field – Varian 600C LA
2	10	2006	Medulloblastoma	Phase I whole CSA + phase II post-fossa boost	55.8 Gy in 31 fractions	Field – Varian 600C LA
3	4	2007	PNET	Whole CSA	35 Gy in 21 fractions	Field – Varian 600C LA
4	35	2009	Ependymoma	Tumour bed + 25 mm margin†	55 Gy in 33 fractions	Field – Siemens Primus LA
5	13	2011	Ependymoma – locoregional recurrence	Phase I whole CSI only‡	39.6 Gy in 22 fractions	Planned volume – TomoTherapy
6	13	2012	Medulloblastoma	Phase I whole CSA + phase II post-fossa boost	60 Gy in 34 fractions (Milan strategy)	Planned volume – TomoTherapy
7	4	2013	Medulloblastoma	Phase I whole CSA + phase II post-fossa boost	54 Gy in 30 fractions	Planned volume – TomoTherapy
8	28	2014	Medulloblastoma	Phase I whole CSA + phase II post-fossa boost	55 Gy in 33 fractions	Planned volume – TomoTherapy
9	16	2014	Ependymoma	Tumour bed + 20 mm margin§	54 Gy in 30 fractions	Planned volume – TomoTherapy
10	32	2014	Medulloblastoma	Phase I whole CSA + phase II post-fossa boost	55 Gy in 33 fractions	Planned volume – TomoTherapy

PNET, primitive neuro-ectodermal tumours; CSA, craniospinal axis; CSI, craniospinal irradiation; LA, linear accelerator.

∗ Clinical target volume to planning target volume margins were 5 mm isotropically for all cases.

† Internal acoustic meatus, jugular foramen and hypoglossal canals were within this 25 mm margin.

‡ Only the phase I dose cube was retrieved from archive for analysis.

§ The Internal acoustic meatus were outside this margin and were therefore not assessed.

### Re-planning and Dose Analysis

**Fig 1:**
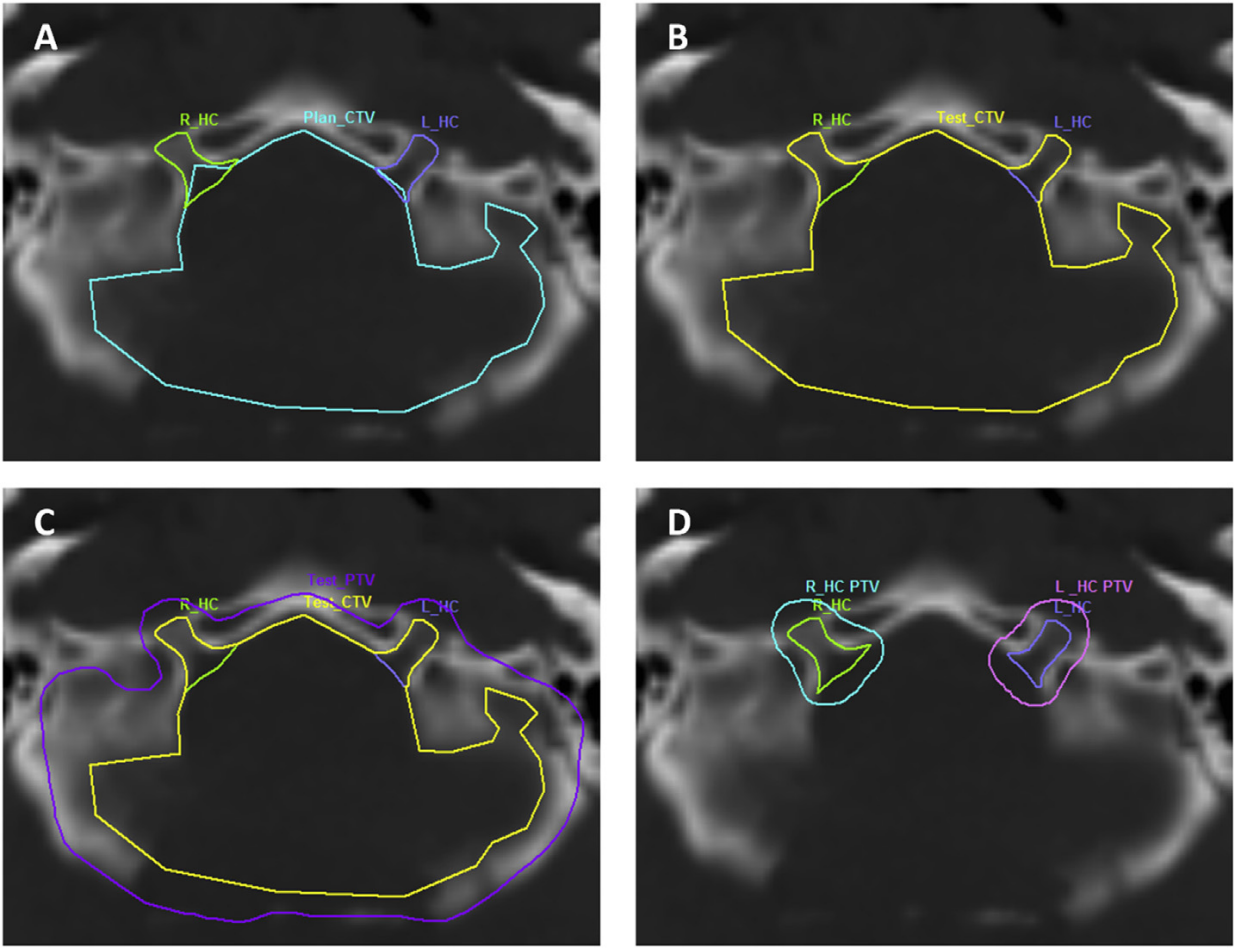
Re-contouring procedure. (A) Contouring right and left hypoglossal canals. (B) Adding to Plan_CTV to create Test_CTV. (C) Growing Test_CTV to Test_PTV. (D) Growing foramen PTVs.

Treatment plans, including DICOM imaging files; structure sets and dose cubes were drawn from archive and reloaded into virtual simulation software (PROSOMA 3.3, OSL, Shrewsbury, UK). Using published anatomical data [17], new contours and target volumes were constructed for each patient. Using bone density windows, a contour was drawn for each individual right and left IAM, JF and HC, to include all possible areas of CSF extension on bone density windows – each volume was drawn and saved as a separate structure; R_IAM, L_IAM, R_JF, L_JF, R_HC, L_HC. The Boolean operator function in PROSOMA was used to fuse these separate volumes with Plan_CTV, and saved as Test_CTV. Test_CTV was grown isotropically by 5 mm to a final planning target volume (Test_PTV) and each foramen CTV was grown by 5 mm to an individual PTV (Figure 1A–D). The treatment plan dose cube was reloaded, individual dose volume histograms (DVHs) were produced for each structure and dosimetric information was recorded.

To compare the quality of treatment plans to the original target volume, we recorded near maximum dose (D_2%_), and median PTV dose, to Plan_PTV. We also calculated dose homogeneity and conformity; homogeneity was calculated using (D_2%_ − D_98%_)/D_50%_ according to ICRU 83 and conformity was calculated using the dice similarity coefficient (DSC), given as 2(A ∩ B)/(A + B), between the Plan_PTV and the 95% isodose line [18], [19].

To compare CTV and PTV dosimetry before and after the addition of posterior fossa cranial nerve foramen volumes, V_95%_ and minimum dose (D_99.9%_) were recorded. For each foramen PTV, V_95%_ and minimum dose (D_98_) were measured. D_99.9_ was chosen for composite PTV and CTV volumes as the Plan_PTV volumes ranged from 280 to 2040 cm^3^ (median 378 cm^3^) and we were interested to see a genuine ‘minimum’ dose. Foramen PTV volumes were generally < 10 cm^3^, thus D_98_ was used.

Anonymised DICOM files and structure sets were encrypted and sent by secure link to a colleague at a collaborating proton beam therapy centre. These files were loaded into their planning system (Raystation 4.7, Raysearch Laboratories AB, Stockholm, Sweden). Pencil beam scanning proton therapy plans using a local cochlea sparing protocol (objectives: maximum dose 50 Gy, mean dose 30 Gy, compromise of PTV, but not CTV, permissible if necessary) were produced for all 10 cases, aiming to adequately cover the original Plan_CTVs. Once these plans were generated, amended target volumes including posterior fossa foramen PTVs, Test_CTV and Test_PTV were loaded, DVH data were derived and returned to the first author for analysis.

### Statistical Analysis

The statistical analysis was performed using IBM SPSS Statistics v 23.0. Paired *t*-tests were used to compare minimum doses to both planned (treated) composite volumes and foramen PTVs with both photon and proton plans. The same method was used to test the significance of adding foramen volumes (to both CTV and PTV) for all three treatment techniques. A significance level of 0.05 was used for all analyses and Bonferroni corrections were used to account for multiple testing.

## Results

### Composite Volumes

Using level 3 reporting metrics [18], both TomoTherapy™ and protons produced higher quality treatment plans than a field-based photon technique. The median dose for field plans ranged from 101.6 to 104.8%. The median doses for TomoTherapy plans were between 99.4 and 100.2% and proton plans ranged from 99.8 to 100.1%. The mean near-maximum dose (D_2%_) for field plans was 108.7% (range 105.5–111%) compared with 101.2% (range 100.8–101.9%) for TomoTherapy and 103.3% (range 101.7–104.8%) for protons. The mean homogeneity index for field plans was 0.17 (range 0.083–0.41) compared with 0.042 (0.022–0.073) for TomoTherapy and 0.072 (0.048–0.091) for protons. Proton plans were the most conformal; the mean DSC was 0.87 for proton plans compared with 0.86 for TomoTherapy and 0.79 for field-based photon plans. Coverage of Plan_PTV (as measured by V_95%_) was generally satisfactory with all treatment techniques. One field plan (case 3) had a V_95%_ of 92% – areas of relative underdosing were laterally over the cranial vault due to build-up.

**Fig 2:**
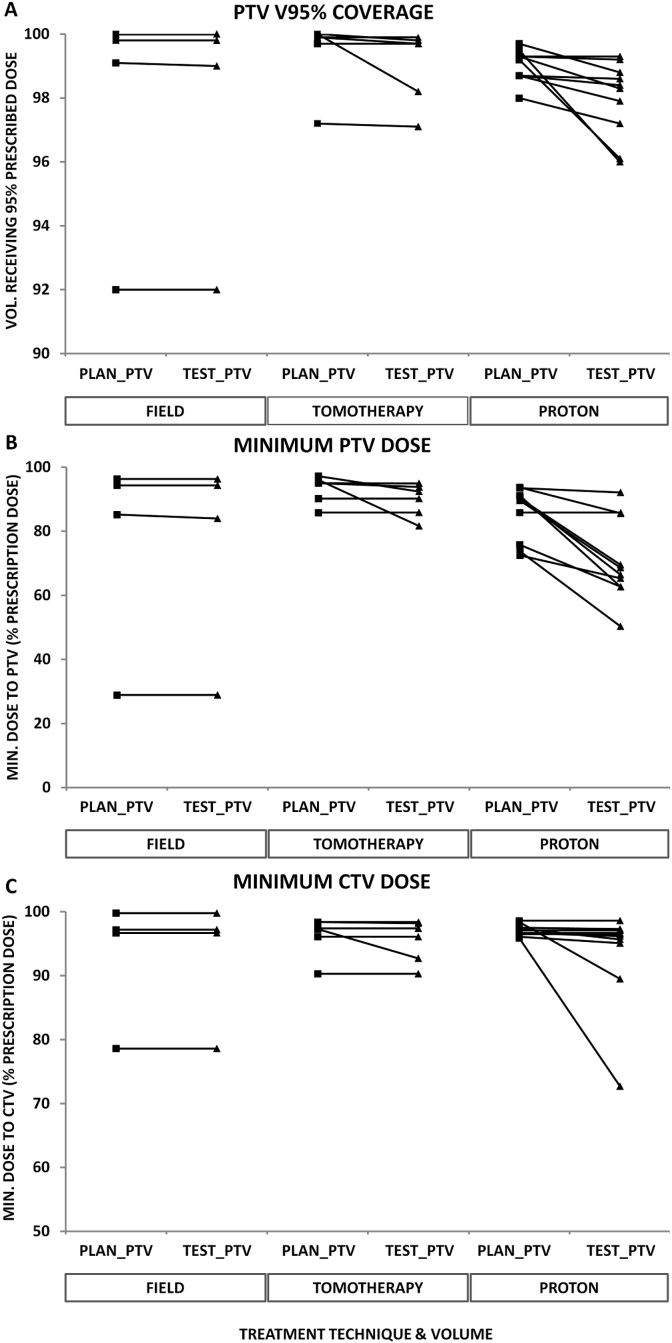
Composite volume coverage, photon and proton plans. (A) PTV V95%. (B) PTV minimum dose (D99.9%). (C) CTV minimum dose (D99.9%).

Figure 2 shows the effect of adding posterior fossa foramen to the dosimetry of both CTVs and PTVs for all three techniques. Adding these structures made no difference to PTV V_95%_, minimum PTV or CTV dose for any of the four field-based treatment plans. These data do show one outlier case (case 3) where target coverage was poor both with and without posterior cranial nerve volumes for reasons described. Adding posterior fossa cranial foramen volumes caused a drop in PTV V_95%_ (100–98.2%) in one of six cases planned and treated with TomoTherapy. Two cases saw a drop in PTV minimum dose of greater than 2% – case 7 (97.2–92.4%) and case 8 (96–81.6%). The mean PTV D_min_ dropped 3.4% from Plan_PTV to Test_PTV for TomoTherapy plans, and this difference was not statistically significant (*P* = 0.20). Only case 8 saw a drop in CTV minimum dose (97.3–92.7%).

The consequences of adding posterior fossa foramen to CTVs were greater with proton plans. The effect on V_95%_ was subtle, but nine of 10 cases saw a lower V_95%_ with Test_PTV than Plan_PTV (mean drop 1.1%, range 0–3.5%). The effect on the minimum PTV dose was more pronounced; half of the proton plans saw >20% drop in minimum dose. The mean Test_PTV minimum dose was 70.9% compared with 85.7% for Plan_PTV. This difference was statistically significant (*P* = 0.001). Dosimetric differences with CTVs were smaller but still apparent. Six of 10 plans saw a drop of <1%, but case 10 saw a drop of 8.9% (98.4–89.5%) from Plan_CTV to Test_CTV, whereas case 8 saw a large drop of 23.2% (95.9–72.7%). The mean minimum CTV dose dropped from 97.1 to 93.5%, but this was not statistically significant (*P* = 0.17).

We compared minimum doses to composite volumes with both photon and proton techniques for all 10 patients. Mean minimum doses for field technique cases (1–4) were 76.2% (photons) versus 91.5% (protons) and 93.2% (photons) versus 97.1% (protons) for Test_PTV and Test_CTV, respectively. Neither difference was statistically significant. For cases 5–10 (TomoTherapy) the respective values for Test_PTV and Test_CTV were 89.8% (photons) versus 70.8% (protons) and 95.5% (photons) versus 91.6% (protons). The *P* value for Test_PTV D_min_ TomoTherapy versus protons was 0.03, suggesting significance. However, once a Bonferroni correction for four significance tests on the Test_volume data is taken into account, α = 0.0125, thus the null hypothesis must not be rejected.

### Individual Foramina

**Fig 3:**
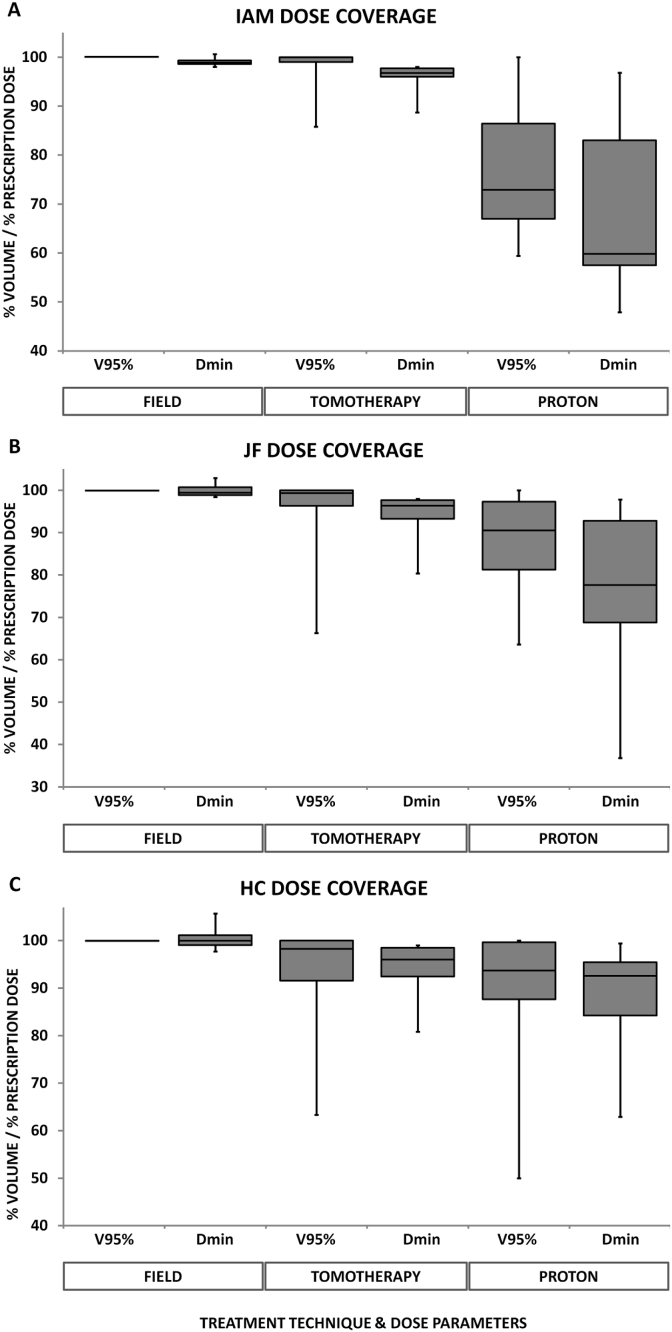
Individual foramen coverage, photon and proton plans. (A) Internal acoustic meatus. (B) Jugular foramen. (C) Hypoglossal canal.

Dosimetry results for the IAM, JF and HC PTVs as separate structures are shown in Figure 3. These structures were well covered by field-based photon plans – the V_95%_ to each foramen PTV was 100% for all field plans. Significant differences between field-photon and proton plan minimum doses were found for all three foramen PTVs (IAM *P* = 0.002, JF *P* = 0.004, HC *P* = 0.003).

TomoTherapy plans covered the IAM well. Only one plan (left IAM, case 7) had a V_95%_ and minimum dose <90% and the mean D_min_ was 95.8%. IAM coverage with protons was less good; the mean D_min_ was 72.3% (range 47.9–96.8%) and this difference was significant (*P* = 0.000). JF coverage with TomoTherapy plans was slightly less good; the mean D_min_ was 94% and the minimum dose was < 95% for 5/12 foramen, the lowest doses being 80.4% and 85.9%, respectively, for left and right JF for case 8. Minimum doses were lower still with proton plans; the mean D_min_ was 78.5% (range 36.8–97.8%). This difference was also significant (*P* = 0.002). The structure with the lowest mean D_min_ with TomoTherapy plans was the HC PTV. D_min_ was <95% for half of these structures and the mean D_min_ was 93.9% (range 80.8–99%). The minimum HC PTV doses were lower with protons (D_min_ mean 89.1%; range 62.9–98.9%). Comparing TomoTherapy and proton plans for HC PTV gives a *P* value of 0.049, but this does not reach significance at the 0.05 level due to Bonferroni correction.

**Fig 4:**
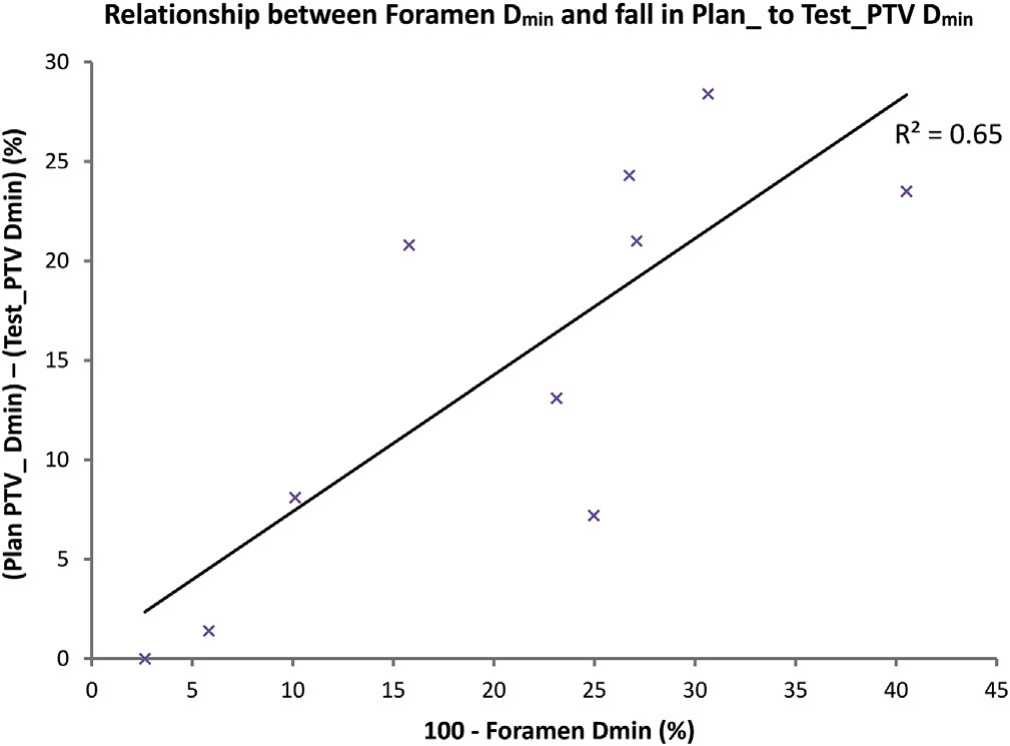
Scatter plot showing the relationship between minimum foramen dose and fall in Plan_PTV to Test_PTV.

**Fig 5:**
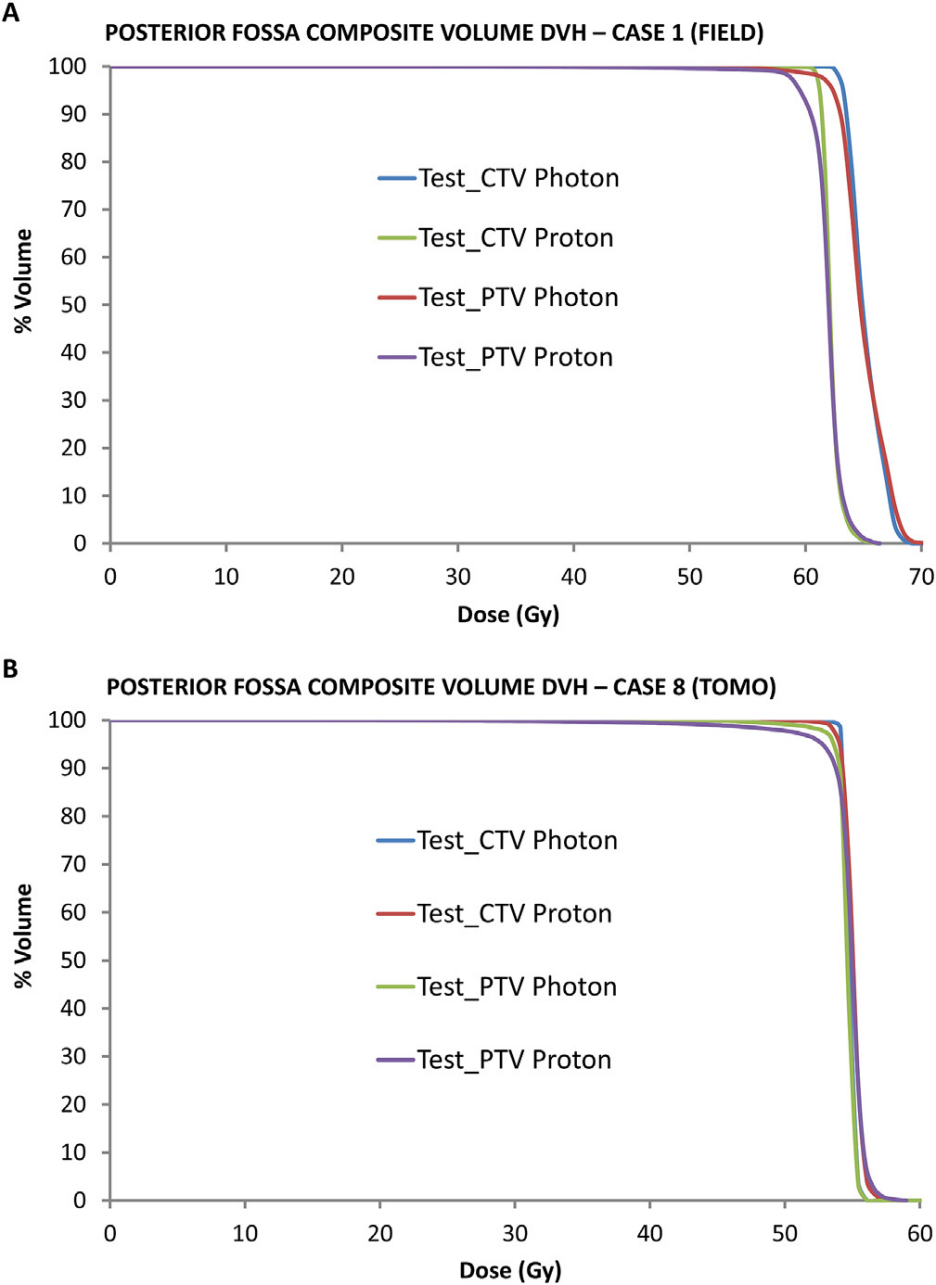
Dose volume histograms for Test_CTV and Test_PTV for photon and proton plans. (A) Case 8: TomoTherapy. (B) Case 1: field.

**Fig 6:**
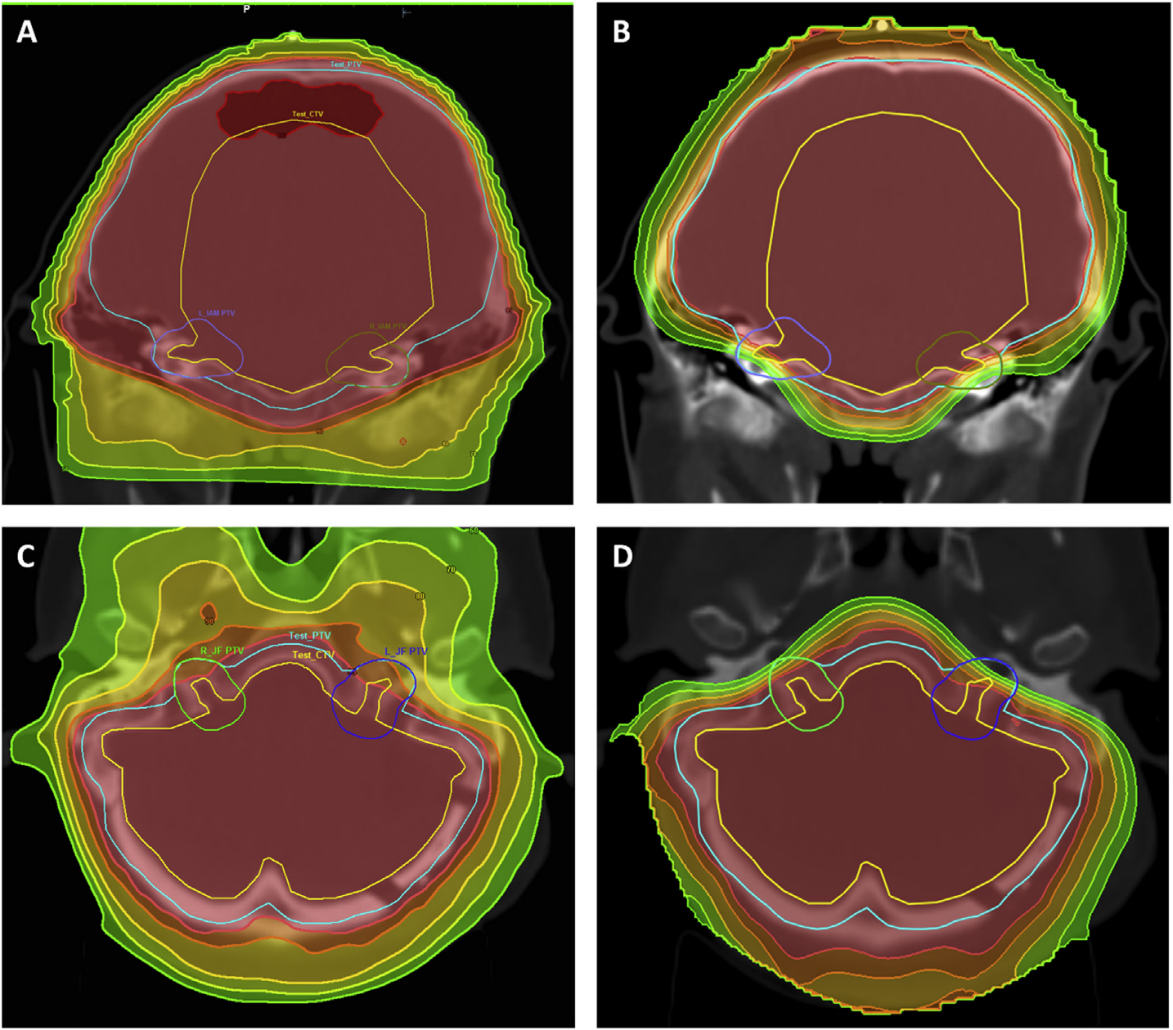
Dose wash for case 1 field (A) and proton (B) plans, case 8 TomoTherapy (C) and proton (D) plans. Colour scheme: dark red 107%, light red 95%, orange 90%, yellow 80%, lime 70%, green 60%.

To prove that low doses to small cranial nerve foramen PTVs affect the dosimetry of much larger Test_CTV and Test_PTVs, we constructed a scatter-plot (Figure 4) comparing the mean D_min_ for all foramina combined, with the drop in D_min_ from Plan_PTV to Test_PTV and found that these parameters are indeed correlated (*r*^2^ = 0.65). We went on to construct DVHs for the two patients with the greatest differences between photon and proton plans; these are shown in Figure 5. For case 1 (Figure 5A), the proton plan covered the CTV well, but there was a noticeable shoulder on the PTV curve due to low foramen doses. Case 8 (Figure 5B) had low foramen doses for both photon and proton plans, but the CTV curve had more of a shoulder with protons than photons and this effect was greater with the PTV curves. The underdosing of foramen PTVs (and therefore Test_PTVs) with proton plans, and to a lesser extent TomoTherapy, is shown visually in Figure 6.

## Discussion

Work on the detailed anatomy of the IAM, JF and HC using fast imaging employing steady-state acquisition (FIESTA) magnetic resonance imaging (MRI) has clearly shown CSF beyond the internal table of the skull, which many clinicians would consider to be a CTV surrogate [17]. This study is the first to investigate the dosimetric implications of failure to include leptomeningeal reflections of posterior fossa cranial nerve foramina in the CTV of CSI treatment plans for medulloblastoma. Specifically, we have shown that this problem is potentially more significant with highly conformal radiotherapy techniques.

There are many advantages to using highly conformal radiotherapy techniques for patients with medulloblastoma, and recent research in this field has focused on how best to use technological advances to spare OARs and reduce long-term toxicity [20], [21], [22], [23]. Helical IMRT solutions spare OARs better than three-dimensional conformal or fixed-field IMRT plans [7] and proton plans offer even greater capacity to reduce dose to most OARs, including eyes, cochlea, thyroid, heart and gonads [10], [11], [24]. Modelling data suggest that these dosimetric advantages will translate to lower cardiac morbidity and second cancer risk [12], [25] and the possible magnitude of these benefits have prompted debate within the community as to whether proton therapy is the only ethical approach to delivering CSI in children [26]. The drive to minimise treatment-related morbidity is both laudable and important, but the primary aim must remain disease eradication for these highly curable tumours.

Our results show that field-based photon plans adequately cover leptomeningeal surfaces in the IAM, JF and HC, regardless of whether or not they are specifically contoured. Helical arc photon therapy plans with TomoTherapy show good coverage of the IAM but potentially significant underdosing of meningeal surfaces in the JF and HC in one of six patients. The effect of increasing conformity is manifestly more obvious with proton plans, where the lowest recorded D_min_ for IAM, JF and HC PTVs were 47.9, 36.8 and 62.9%, respectively, and this effect is well visualised in Figure 6.

We sought to address the relevance of low doses to small components of a much larger structure, on overall dosimetry of the larger structure. The data in Figure 2 show that adding posterior fossa foramina to composite CTV and PTV for proton plans made a noticeable difference to the minimum dose. For proton plans, there seems to be a relationship between cases that saw the lowest foramen doses and those in which differences between Plan and Test CTV and PTV doses are apparent. This same effect is also seen in the DVHs in Figure 5.

Recent data on medulloblastoma relapse suggest that the posterior fossa remains a high-risk site, despite modern radiotherapy protocols that boost this region. One single institution series (median age 7 years, range 0–50, *n* = 106) found an overall relapse rate of 27%, of which 41% involved the posterior fossa [27]. Another study looking at paediatric patients from two centres (one USA, one Canadian, 89 medulloblastoma) found overall relapse rates of 29%, with 27% of these in the posterior fossa [28]. A study that looked at 20 adult patients found that 71% of the recurrence in their series involved the posterior fossa [29]. To the best of our knowledge, however, there are no data specifically pertaining to marginal recurrence of medulloblastoma in the structures discussed in this study. Furthermore, an early report directly comparing outcomes of patients undergoing treatment with photon and proton techniques showed no significant differences between techniques [30].

Our data show that compared with field-based photon CSI plans, TomoTherapy and proton plans give lower median and maximum doses to the PTV, better target conformity and dose homogeneity within that target. Nonetheless, we have also shown that the CSF and meninges found in the foramina of the posterior fossa are underdosed with highly conformal radiotherapy techniques, unless they are specifically contoured, and that this may have a small but noticeable effect on the overall dosimetry of the treatment plan.

The limitations of this study are firstly that it was based on a small and heterogeneous case series and to increase sample size two patients who had not undergone CSI were included. Importantly however, the CTVs used in these cases closely resembled a typical phase II for a medulloblastoma plan and the margins used extended beyond their skull base constraints into skull base foramina. Because of its design, we have not been able to directly compare these dosimetric effects in field-based and TomoTherapy plans. In conducting this study, we found that there was inconsistency in the way that CTVs were contoured around these structures. In some cases, the Plan_CTV already included part or all of the IAM and JF. Although this makes the point that there is a need for anatomical and dosimetric data to guide segmentation, it may have weakened the findings of our study.

Finally, this study has not looked at doses to OARs. The proton plans in this study were generated with a cochlea-sparing protocol, whereas the photon plans were not. This may slightly reduce the strength of direct plan comparison, but accurately reflects the current clinical dilemma. Published FIESTA MRI data and a previous surgical study prove that dura and CSF are present to the fundus of the IAM [17], [31]. Equally, the risk of sensorineural deafness rises sharply with doses above 40–45 Gy [32], [33]. It has been shown that three-dimensional conformal and IMRT photon techniques can spare the cochlea relative to field-based photon plans [34] and that proton plans are better still [24], [35]. These dosimetric advantages seemingly translate to clinical benefit, which may in turn have long-term economic benefit [36], [37]. However, these studies do not explicitly describe how the CTV was constructed around the IAM. Images from St Clair *et al.* [24] suggest that the IAMs were not included in the CTV in this study. In the era of protons, cochlea sparing is technically possible, but there is ongoing debate within the neuro-oncology community as to how this should be prioritised, particularly for CSF-positive medulloblastoma.

Our data show that however conformal the radiotherapy technique, it is not possible to simultaneously treat meninges/CSF in the IAM and keep the cochlea dose below tolerance. We suggest that the full IAM should be contoured as CTV. However, oncologists must weigh the balance between cure and morbidity and prioritising cochlea sparing for lower risk patients by using exclusion structures during the planning process may be justified. Promising work on molecular risk stratification may help to inform such decisions [38] and there is a clear need for careful long-term follow-up of patients treated with CSI.

## Conclusions

This study has shown that the meningeal reflections and CSF surrounding cranial nerves VII–XII as they pass into posterior fossa skull base foramina are underdosed by highly conformal radiotherapy techniques, unless they are specifically included in the CTV. Such findings may have implications for tumour control and it is essential to monitor relapse patterns for patients treated for medulloblastoma. We have not addressed the impact that such inclusion would have on dose to OARs, specifically the cochlea, but this is a subject for further work.
